# Characterization of SIBLING Proteins in the Mineralized Tissues

**DOI:** 10.3390/dj10080144

**Published:** 2022-08-04

**Authors:** Sandeep Dab, Nancy Abdelhay, Carlos Alberto Figueredo, Seema Ganatra, Monica Prasad Gibson

**Affiliations:** 1 School of Dentistry, Faculty of Medicine and Dentistry, University of Alberta, Edmonton, AB T6G1C9, Canada; 2 Faculty of Dentistry, Alexandria University, Alexandria 5423012, Egypt

**Keywords:** dentin sialophosphoprotein, non-collagenous proteins, bone, craniofacial development

## Abstract

The SIBLING proteins are a family of non-collagenous proteins (NCPs) previously thought to be expressed only in dentin but have been demonstrated in other mineralized and non-mineralized tissues. They are believed to play vital roles in both osteogenesis and dentinogenesis. Since they are tightly regulated lifelong processes and involve a peak of mineralization, three different age groups were investigated. Fifteen wild-type (WT) mice were euthanized at ages 1, 3, and 6 months. Hematoxylin and eosin staining (H&E) was performed to localize various microscopic structures in the mice mandibles and tibias. The immunostaining pattern was compared using antibodies for dentin sialoprotein (DSP), dentin matrix protein 1 (DMP1), bone sialoprotein (BSP), and osteopontin (OPN). Immunostaining of DSP in tibia showed its most noticeable staining in the 3-month age group. DSP was expressed in alveolar bone, cellular cementum, and PDL. A similar expression of DMP1 was seen in the tibia and dentin. BSP was most noticeably detected in the tibia and acellular cementum. OPN was mainly expressed in the bone. A lower level of OPN was observed at all age groups in the teeth. The immunostaining intensity was the least detected for all proteins in the 6-month tibia sample. The expression patterns of the four SIBLING proteins showed variations in their staining intensity and temporospatial patterning concordant with skeletal and dental maturity. These findings suggest some role in this tightly regulated mineralization process.

## 1. Introduction

Morphologically and functionally, the body is composed of a complex organization of specialized tissues working together for specific functions [1]. Bone is one specialized connective tissue that provides structural strength and rigidity to the body [1]. The bone matrix has both organic and inorganic components. The organic component of the bony extracellular matrix (ECM) comprises approximately 20% of the bone weight. The majority of ECM (≈90%) is composed of collagen type 1, providing flexibility to the bone [1,2]. The collagen fibers, secreted by specialized cells, form a scaffold that is suspended in ground substance. The inorganic hydroxyapatite crystals which contribute 65–70% of bone wet weight surround the collagen fibers of the organic matrix [1,3]. A small percentage of this organic ECM represents highly multi-functional non-collagenous proteins (NCPs) [4]. Controlled mineralization of the ECM components occurs under the influence of various molecules including NCPs [3,5,6].

Bone, dentin, and cementum share relatively similar composition and development mechanisms. The precursor cells for bone, dentin, and cementum produce a distinct set of extracellular matrix molecules composed predominantly of type I collagen fibrils [7]. Unique to the bone, cementum, and dentin formation processes is the biomineralization of the unmineralized proteinaceous matrix. [7,8]. Since both dentinogenesis and odontogenesis involve the conversion of unmineralized structures to mineralized products, these processes strongly suggest the sharing of critical steps [9]. The control of these mechanisms must involve the processing and activation of certain molecules [10]. Pathological disruption of this regulation may lead to changes in the mineralization of matrices. This disruption of regulation is phenotypically exemplified in pathological conditions such as dentinogenesis imperfecta and osteogenesis imperfecta where mutations lead to dentin and bone mineralization defects [11,12].

One such NCP is the SIBLING (Small-Integrin-Binding Ligand, N-linked Glycoprotein) family (a calcium-binding family), which has five proteins: dentin sialophosphoprotein (DSPP), which undergoes post-translational modification to be cleaved into dentin sialoprotein (DSP) and dentin phosphoprotein (DPP); dentin matrix protein 1 (DMP1), bone sialoprotein (BSP), osteopontin (OPN), and matrix extracellular phosphoglycoprotein (MEPE) [13,14,15,16].

The SIBLING proteins have been associated with biomineralization as enhancers and/or inhibitors [17,18]. It has been previously reported that mutations in the SIBLING family genes are associated with abnormalities in their phenotypic expression. Two members, DSPP and DMP1, have been directly associated with some human genetic diseases. Phenotypically, these diseases present as alterations manifesting as hypomineralization of bone and dentin [19,20]. It has been shown that disruption of the biomineralization process leads to altered morphotype and phenotype. The SIBLING family of proteins displays differential temporospatial expression profiles in tissues where post-translational modifications are important. Together, they are believed to actively orchestrate hydroxyapatite mineralization and crystal growth. The SIBLING proteins have been widely evaluated individually, and their key roles in the process of biomineralization have been characterized [8,13]. These pieces of evidence direct us to the need to explore further pathways critical to biomineralization and their involvement. Therefore, the objective of this study was to characterize the expression of SIBLING proteins in the long bones and periodontium of 1-, 3-, and 6-month-old mice. 

## 2. Materials and Methods

### 2.1. Design

Fifteen wild-type (WT) mice (C57BL/6J—The Jackson Laboratory; Bar Harbor, ME, USA) were investigated. Five hemi-mandibles and five tibias each for ages 1, 3, and 6 months old were randomly selected. This research followed protocols approved by the Animal Welfare Committee at the University of Alberta Health Sciences (ACUC Committee approval #AUP0002086). The specimens for histology and immunohistochemistry were decalcified in 15% EDTA (pH 7.4) at 4 degrees [21]. 

### 2.2. Hematoxylin and Eosin (H&E)

Paraffin was used to embed decalcified samples and 5 m thick sections were sliced in a mesiodistal direction. H&E staining was performed following the protocol developed by Sigma-Aldrich (Sigma-Aldrich, St. Louis, MI, USA) [22].

### 2.3. Immunohistochemistry (IHC) Staining

IHC staining was completed to investigate the distribution and expression pattern of DSPP, DMP1, BSP, and OPN. MEPE was not part of our project since its expression is only present until day 9 post-natal [23,24]. IHC staining of DSPP/DSP, an anti-DSP monoclonal antibody referred to as anti-DSP-2C12.3 [25], was used at a dilution of 1:800. IHC staining of DMP1 and anti-DMP1-C-8G10.3 MAb recognizing the COOH- terminal region of DMP1 [26] was used at a dilution of 1:400. For BSP, polyclonal rabbit anti-mouse (KLH conjugated synthetic peptide IgG derived from mouse bone sialoprotein) and for OPN, polyclonal (purified recombinant mouse OPN) goat anti-mouse were used as primary antibodies at a concentration of 1:200. Concentration of the primary antibody was selected to minimize background staining according to previous research. The negative control for DSP and DMP-1 was normal mouse immunoglobulin G (IgG) secondary antibody, and the negative control for BSP and OPN was normal rabbit anti-goat serum [27].

The secondary antibody for DSP and DMP-1 was biotinylated horse anti-mouse IgG (Vector Laboratories, Burlingame, CA, USA) at 1:200 concentration. The secondary antibody for BSP and OPN was biotinylated goat anti-rabbit IgG (Vector Laboratories) at 1:200 concentration. Rabbit IgG isotype control (bs-0295p-Bioss Inc., Woburn, MA, USA) was used as a control for staining [25,26,27]. IHC protocols were conducted with the Vectastain ABC kit and 3,3′ diaminobenzidine DAB kit (Vector Laboratories; Burlingame, CA, USA) according to the manufacturer’s recommendations for color visualization of immunoreactivity. Methyl green was used for counterstaining.

## 3. Results

### 3.1. Long Bones

#### IHC Staining

**Figure 1:**
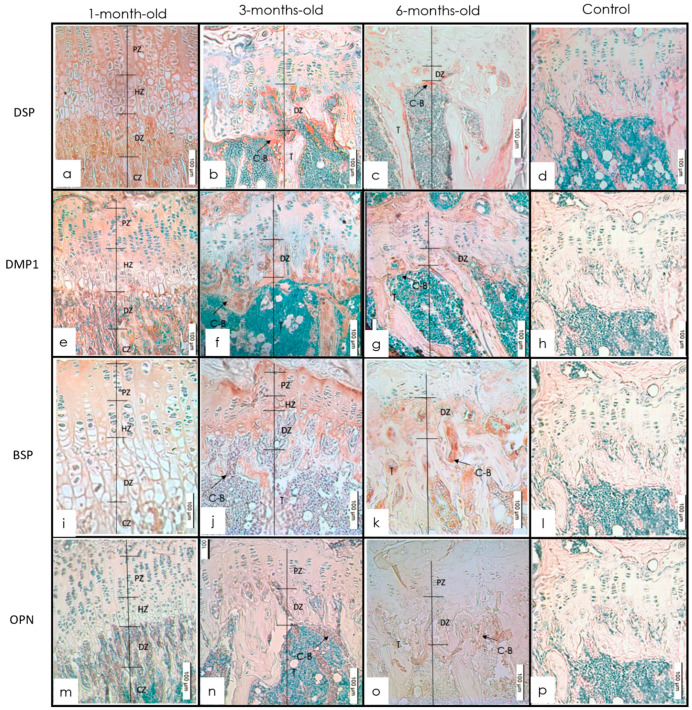
(**a**–**d**) Expression of staining intensity of DSP at 1, 3, and 6 months in metaphysis and control with anti-DSP (IHC 20×). Intensity increased from 1 to 3 months followed by a decrease between 3 and 6 months (source—IHC Dab). (**e**–**h**) IHC staining of metaphysis and growth plate in 1-, 3-, and 6-month-old mice with anti-DMP1 (IHC 20×). Intensity increased from 1 to 3 months and then decreased between 3 and 6 months. (**i**–**l**) IHC of metaphysis and growth plate in 1-, 3-, and 6-month-old mice with anti-BSP (IHC 20×) Most noticeable staining was found at 3 months. (**m**–**p**) IHC staining of metaphysis and growth plate in 1-, 3-, and 6-month-old mice with anti-OPN (IHC 20×). The intensity increased from 1 to 3 months followed by a decrease between 3 and 6 months.

**Figure 2:**
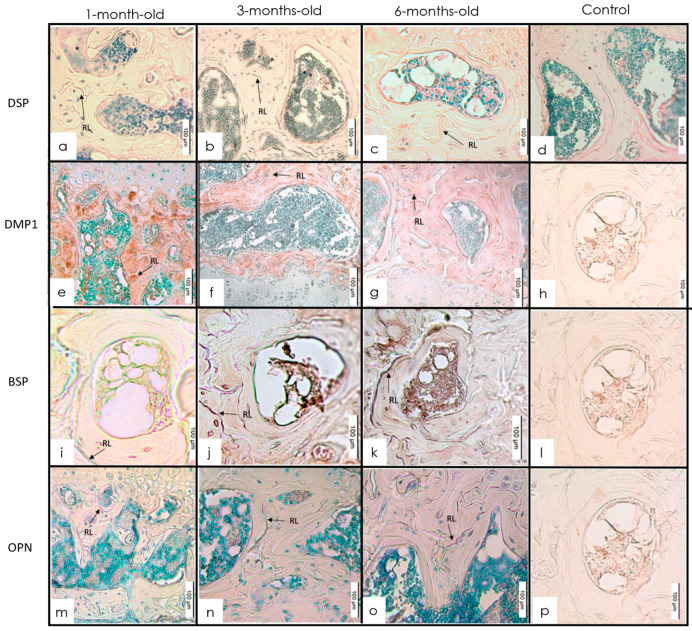
(**a**–**d**) Expression of staining intensity of DSP at 1, 3, and 6 months in the epiphysis with anti-DSP (IHC 20×). Intensity increased from 1 to 3 months followed by a decrease between 3 and 6 months. (**e**–**h**) IHC staining with anti-DMP1 in the epiphysis of 1-, 3-, and 6-month-old mice (IHC 20×). Intensity decreased from 1 to 3 months followed by a further decrease between 3 and 6 months. The most noticeable staining was seen at 1 month. (**i**–**l**) Expression of staining intensity of BSP in epiphysis of 1-, 3-, and 6-month-old mice. IHC of epiphysis in 1-, 3-, and 6-month-old mice with anti-BSP (IHC 20×). Intensity increased from 1 to 3 months and decreased between 3 and 6 months. (**m**–**p**) Expression of staining intensity of OPN at 1, 3, and 6 months in the epiphysis. (IHC 20× intensity increased from 1 to 3 months and decreased between 3 and 6 months.)

The IHC of the tibia from 1-, 3-, and 6-month-old mice showed that each of the four SIBLING proteins had a distinct expression pattern in the different mineralized tissues. The signal for DSP spread diffusely throughout the growth plate and metaphysis at 1 month. Expression was present at epiphysis and in areas of transformation into trabeculae. At 3 months, the expression pattern of DSP was present at the calcifying front (CZ) of the growth plate. Notable expression was seen in the degenerating chondroblasts at the cartilage–bone interface. At 6 months, expression was detected at the epiphysis and the calcification front of the primary ossification center (Figure 1a and Figure 2a). There was a positive expression of DMP1 throughout the growth plate of 1-month-old mice and particularly at the interface of the degenerating chondroblasts and newly forming bony trabeculae. The DMP1 expression was at the CZ interface of mice of all ages. A noticeable signal for DMP1 was visualized in the bone surrounding marrow spaces in the epiphysis. The expression pattern for DMP1 showed a further decline at 6 months (Figure 1b and Figure 2b). At 1 month, the tibial growth plate and metaphysis showed diffuse BSP expression. This was followed by an increase in expression at 3 months. The expression of BSP in the growth plate was especially concentrated in the resting zone (RZ), proliferating, and hypertrophic layers of the growth plate. This expression was different from other proteins which were concentrated along the calcification border at the chondrocyte–bone interface. The expression was mainly localized in the hypertrophic and degenerating chondrocytes as well as the calcification zone. Similar to the metaphysis, BSP expression in the epiphysis showed increased staining intensity from 1 to 3 months followed by a decline between 3 and 6 months (Figure 1c and Figure 2c). OPN was expressed in the calcification zone of the growth plate and metaphysis of the tibia at all ages. There was a slight increase in staining intensity of OPN from 1 to 3 months followed by a decrease from 3 to 6 months. A similar expression pattern was observed in the epiphysis within the same age groups (Figure 1d and Figure 2d).

### 3.2. Alveolar Bone

#### 3.2.1. H&E Staining

**Figure 3:**
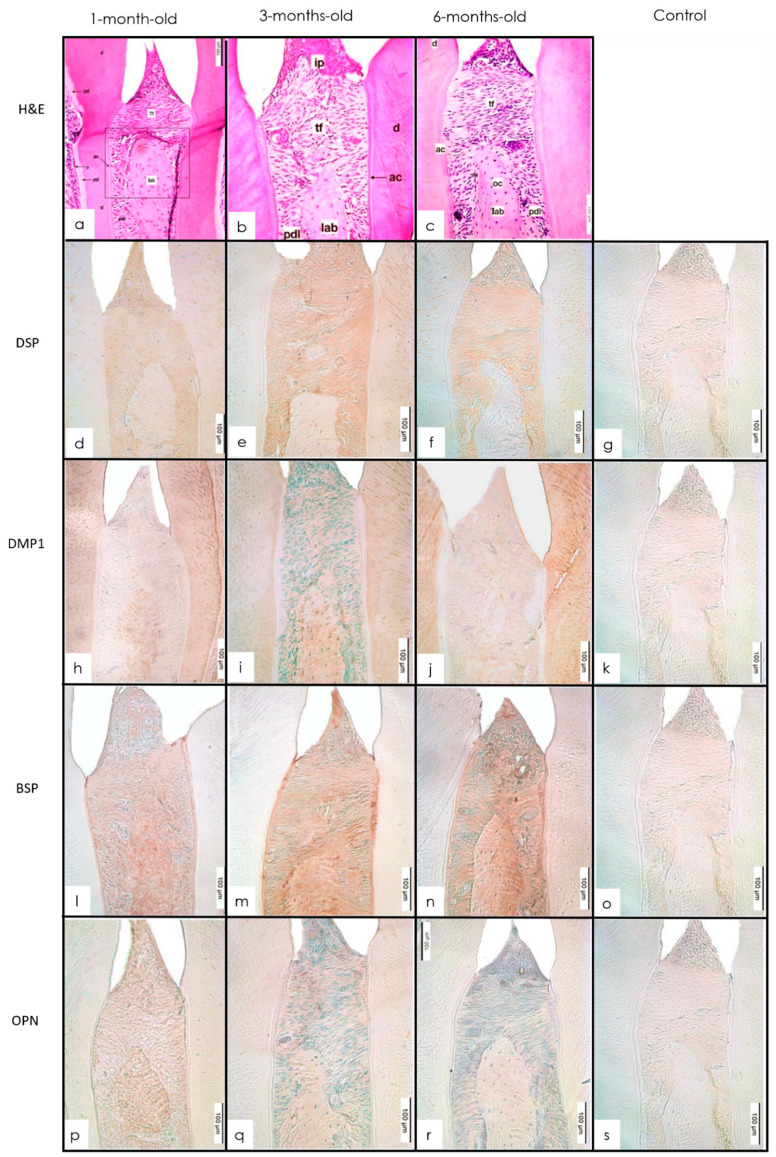
(**a**–**c**) H&E molar showing interdental bone 1-, 3-, and 6-month-old mice (H&E 20×). The interproximal bone is well-developed in all age groups. d—dentin; pd—predentin; o—odontoblastic layer; p—pulp; fb—furcal bone; pdl—periodontal ligament; tf—transeptal fibers; Iab—interproximal alveolar bone. (**d**–**g**) Expression of staining intensity of DSP at 1, 3, and 6 months in interproximal bone with anti-DSP (IHC 20×). Intensity decreased from 1 to 3 months followed by a slight decrease between 3 and 6 months. (**h**–**k**) IHC staining of interproximal alveolar bone in 1-, 3-, and 6-month-old mice with anti-DMP1 (IHC 20×). There was an intensity change from 1 to 3 months. (**l**–**o**) IHC staining of interproximal alveolar bone in 1-, 3-, and 6-month-old mice with anti-BSP (IHC 20×). There was minimal change in intensity from 1 to 3 months followed by an increase between 3 and 6 months. (**p**–**s**) IHC staining of interproximal alveolar bone in 1-, 3-, and 6-month-old mice with anti-OPN (IHC 20×). Intensity decreased from 1 to 3 months and slightly increased between 3 and 6 months.

**Figure 4:**
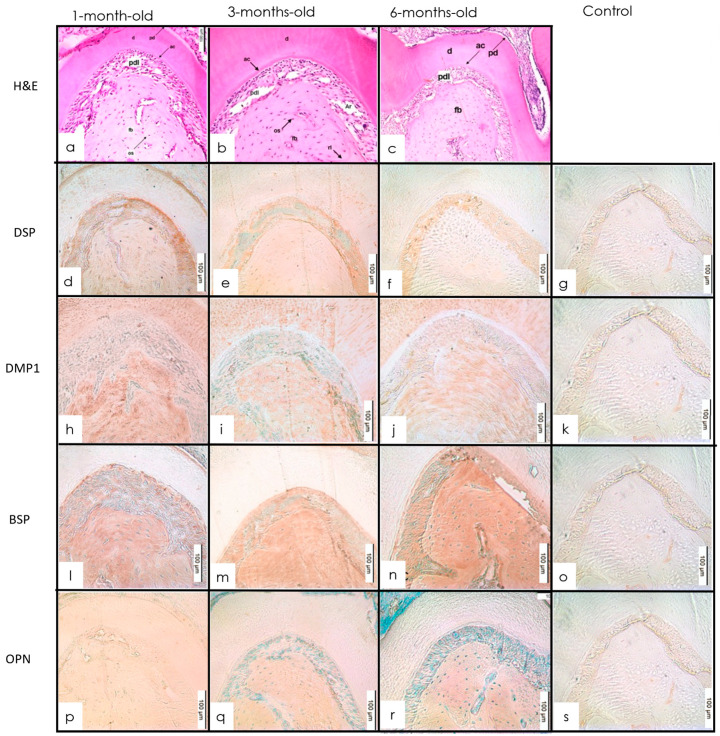
(**a**–**c**) H&E molar showing furcation alveolar bone 1-, 3-, and 6-month-old mice (H&E 20×). The furcation bone is well-developed in all age groups. d—dentin; pd—predentin; o—odontoblastic layer; p—pulp; fb—furcal bone; pdl—periodontal ligament; tf—transeptal fibers; Iab—interproximal alveolar bone. (**d**–**g**) Expression of staining intensity of DSP at 1, 3, and 6 months in furcation bone with anti-DSP (IHC 20×). Intensity decreased from 1 to 3 months followed by a slight decrease between 3 and 6 months. (**h**–**k**) IHC staining of furcation alveolar bone in 1-, 3-, and 6-month-old mice with anti-DMP1 (IHC 20×). Intensity decreased from 1 to 3 months and decreased further between 3 and 6 months. (**l**–**o**) IHC staining of furcation alveolar bone in 1-, 3-, and 6-month-old mice with anti-BSP (IHC 20×). Intensity decreased slightly from 1 to 3 months followed by an increase between 3 and 6 months. (**p**–**s**) IHC staining of furcation alveolar bone in 1-, 3-, and 6-month-old mice with anti-OPN (IHC 20×). Intensity decreased from 1 to 3 months followed by an increase between 3 and 6 months.

The crowns of the teeth had fully developed dentin with normal thickness of predentin and a layer of odontoblasts lining the pulp chamber near the unmineralized dentin matrix. A well-developed interdental papilla was seen with transseptal fibers crossing the interdental alveolar bone inserting into the cementum on either side. The PDL and pulp were well-developed and organized tissue structures (Figure 3a and Figure 4a). The predentin layer maintained a relatively constant width in all three age groups (Figure 3a and Figure 4a).

#### 3.2.2. IHC Staining

Interproximally, the interdental bone showed a decrease in the expression of DSP from 1 to 3 months (Figure 3b). In the interproximal bone, DMP1 was expressed at 1 month followed by an increase in the expression at 3 months. This was followed by a decrease in expression from 3 to 6 months falling below 1-month levels (Figure 3c). In the interproximal region, BSP showed a slight increase in its expression from 1 to 3 months followed by an increase from 3 to 6 months (Figure 3d). The interproximal bone showed a similar expression pattern of OPN with a decrease from 1 to 3 months followed by an increase from 3 to 6 months (Figure 3e). In the furcation region, there was a decrease in the level of expression from 1 to 3 months followed by a further moderate decrease in the expression of DSP from 3 to 6 months. The expression of DMP1 in the furcation was high at 1 month followed by a slight decrease at 3 months. Within the furcal bone, BSP showed a slight decrease from the 1- to 3-month group followed by an increase in expression at 6 months. OPN expression in the furcal bone seemed to remain consistent with a mild increase from 1 to 3 months followed by a decrease from 3 to 6 months (Figure 4b–e).

### 3.3. Cementum

#### 3.3.1. H&E Staining

**Figure 5:**
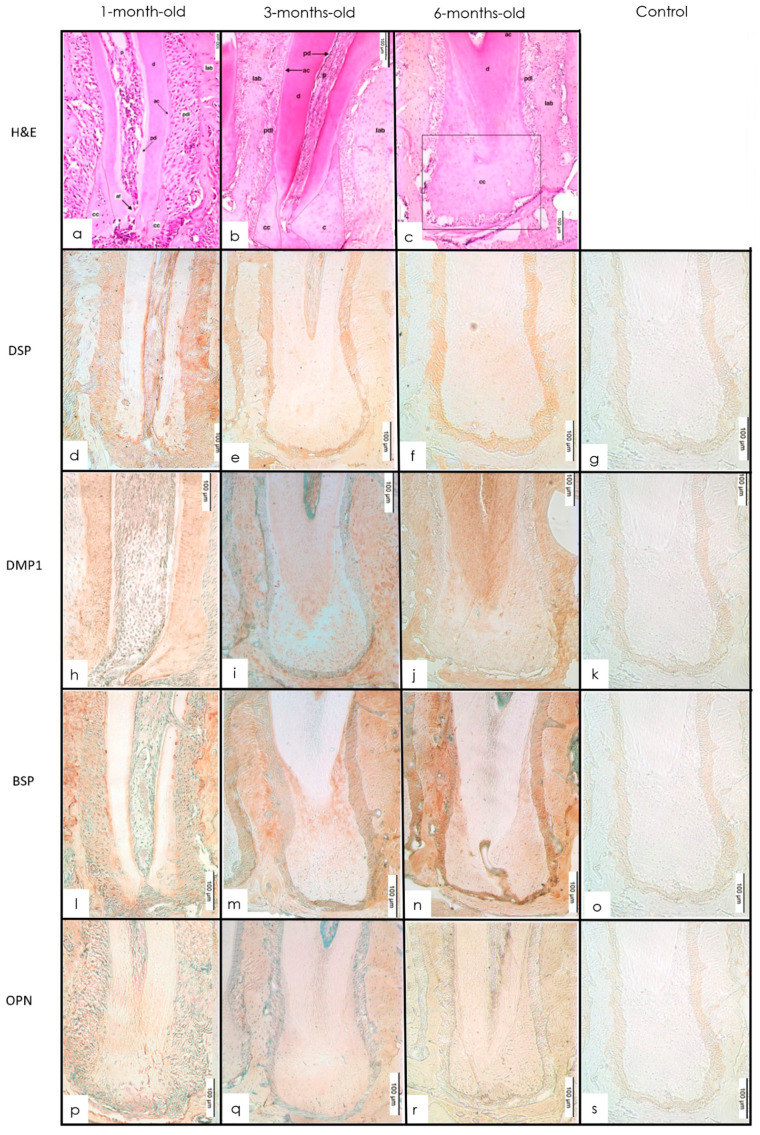
(**a**–**c**) H&E molar showing cementum at 1-, 3-, and 6-month-old mice (H&E 20×). The cementum and apical bone are well-developed in all age groups and the root apex continues developing with abundant cellular cementum. d—dentin; pd—predentin; o—odontoblastic layer; p—pulp; fb—furcal bone; pdl—periodontal ligament; tf—transeptal fibers; Iab—interproximal alveolar bone; cc—cellular cementum; ac—acellular cementum; af—apical foramen. (**d**–**g**) Expression of staining intensity of DSP at 1, 3, and 6 months in cellular cementum and PDL of the tooth root. With anti-DSP (IHC 20×). Intensity decreased from 1 to 3 months followed by a slight increase at 6 months. (**h**–**k**) IHC staining of cellular cementum and PDL in tooth root in 1-, 3-, and 6-month-old mice with anti-DMP1 (IHC 20×). The intensity increased from 1 to 3 months followed by an increase between 3 and 6 months. (**l**–**o**) IHC staining of cellular cementum and PDL in tooth root in 1-, 3-, and 6-month-old mice with anti-BSP (IHC 20×). The intensity increased slightly from 1 to 3 months followed by an increase between 3 and 6 months. (**p**–**s**) IHC staining of cellular cementum and PDL in tooth root in 1-, 3-, and 6-month-old mice with anti-OPN (IHC 20×). Insignificant changes in intensity were seen between 1 and 3, and 3 and 6 months, respectively.

There was a thin layer of cellular cementum at the apices. At 3 and 6 months, the root structure was well developed. A thin layer of acellular cementum lined the cervical two-thirds of the roots. A reactionary dentin zone stained intensely with an accentuated horizontal incremental line pattern was detected. The predentin layer maintained a relatively constant width in all three age groups (Figure 5a).

#### 3.3.2. IHC Staining

DSP was expressed in the PDL around the root. At the root apex, there was a decrease in the expression from 1 to 3 months, whereas from 3 months to 6 months, there was a slight increase in the expression of DSP. The cellular cementum at the apex of the root showed DSP staining albeit with less intensity and no staining of the acellular cementum was observed in all age groups. The roots showed a positive signal for DMP1 within the dentin, periapical PDL, and cellular cementum. Strong BSP signals were observed in the interdental alveolar bone, furcation bone, and acellular cementum. Signals for cellular cementum were localized to the cervical and apical portions of the root. In the apical third of the root, BSP was expressed in the dentin, cellular cementum, and the PDL. Within the cellular cementum and PDL, there was a slight increase in expression from 1 to 3 months followed by an increase in the expression of BSP from 3 to 6 months in the root. There was no detected staining of OPN in the acellular cementum in any age group. A similar pattern of expression was observed in the root with a slight increase from 1 to 3 months followed by a decrease from 3 to 6 months (Figure 5b–e).

## 4. Discussion

The differences of distribution and level of expression of SIBLING proteins in the bone and mandible at three different age groups were investigated thoroughly. The dynamic process of conversion of predentin and osteoid into mineralized matrices and the maintenance of a uniform layer indicates tight regulation of complex crosstalk. This happens among cells and several components of ECM including type I collagen, NCPs (such as SIBLING proteins), and proteoglycans [28]. A better understanding of the pathways of biomineralization might be beneficial to different research areas, including regenerative endodontics [29,30].

The newly forming trabeculae and growth plate are responsible for a rapid increase in the length of the bone. The increase in the rate of mineralization could be attributed to the further skeletal development and increase in compressive function of the tibia from 1 to 3 months. On attaining skeletal maturity at 6 months, there is a concomitant decrease in the rate of mineralization.

In the current study, DSP was localized at the bone-forming front. This is consistent with previous studies that showed stronger signals for DSPP mRNA adjacent to the growth plate [6,31]. At 1 month, C57BL/6 mice tibia were still developing with a high rate of osteoid formation, and the mineral content had not peaked. During the rapid phase of development, there is a continual increase in the amount of osteoid until 3 months of age. There is also a concomitant shift in the rate of conversion of osteoid into the mineralized matrix at the cartilage–bone interface. The decreased osteoid volume was accompanied by an increase in trabecular thickness and mineral density [25,32]. At 6 months, the C57BL/6 mice reach their peak bone mass, scant new bone is formed, and reduced remodeling occurs compared to 3 months. The reduction in expression of DSP in the tibia at 6 months happened at the same time as the reduction in the size of the growth plate and skeletal maturity. This reduced expression may follow the reduced rate of mineralization and remodeling of the trabeculae with age [26]. The expression of anti-DSP antibody in the alveolar bone, cellular cementum, and the periodontal ligament is consistent with the previous study by Huang showing DSP to be primarily present in the ECM and organic extract [17]. Its presence might facilitate the nucleation of HA along the collagen fibril converting the predentin to dentin [8,33,34]. It has also been shown that the NH2 terminal (DSP) may play an inhibitory role by preventing the premature mineralization of predentin by serving as an antagonist to DPP’s mineralizing-accelerating action. It has been shown that DSPP is secreted by a variety of cells and is related to the initial formation of the periodontal ligament [25]. The detection of DSP expression in PDL, cellular cementum, dentin, and alveolar bone is consistent with previous findings of DSP confirmed by in situ hybridization and IHC [35]. A lack of expression in acellular cementum is suggestive that this material’s novel formation/homeostasis pattern is different from cellular cementum [36]. 

Similar to DSPP, DMP1 is also present in the amino and carboxy fragments in bone and dentin ECM [34]. In our IHC experiment, we used the anti-DMP1 C-terminal which demonstrated that the majority of DMP1 was present in the ECM of bone and dentin [37]. This terminal, due to its high negative charge and phosphorylation, has been implicated as an initiator in the mineral deposition by acting as a nucleator for hydroxyapatite [37]. The presence of type I collagen phosphorylated DMP1 C-terminal fragments in bones of rats has been shown by Tartaix et al. to accelerate nucleation of hydroxyapatite [38]. Localization of expression in the degenerating hypertrophic chondrocytes indicates it may have some regulatory function. The ECM of degenerating chondrocytes undergoes vascular invasion, resorption by chondroclasts, and deposition of osteoid. This is followed by osteoblastic/osteoclastic remodeling of osteoid into mineralized bone via the nucleation of hydroxyapatite under the influence of some molecules [39]. Yang showed a significantly increased osteocytic expression of DMP1 when subjected to mechanical loading [40]. Previous studies suggested that the secondary ossification center (SOC) develops early between 5 and 14 days in C57BL/6 mice and hinted at the importance of SOC in protecting the growth plate. It has also been previously shown that DMP1 expression increases with compressive mechanical loading in vitro and in vivo [17]. This suggests that the mineralization of the bone in the epiphysis starts early, contributing to an increase in the width of bone at the epiphyseal end and continues to decline thereafter. Therefore, the higher expression of DMP1 at 1 month may portray its pattern following earlier epiphysis development [41]. 

BSP antibody has been shown to have broader interactions with various components of the ECM [42]. BSP is least abundant in the unmineralized matrix, moderately present in the decalcified exposed bone extract, and most abundant in the mineralized phase tightly bound to apatite crystals [43]. Since BSP was shown to have a binding affinity for type I collagen, its expression was diffuse at 1 month of age. While BSP has been shown to have bonded to both collagen and hydroxyapatite [43], at 3 months, active conversion of osteoid into mineralized bone was detected. Its expression in all layers of the growth plate may also indicate that BSP might regulate the rate of mitosis of chondrocytes in the resting and proliferating zones. Its presence in unmineralized predentin, growth plate, and acellular cementum shows BSP may have a higher binding affinity for GAGs such as chondroitin sulfate and proteoglycans [44]. 

The pattern of expression of BSP in alveolar bone is different from long bones. A possible explanation is that the alveolar bone forms by intramembranous ossification, which is a process that is different from endochondral ossification. Moreover, at 3 months, the molars are fully erupted and undertake increased masticatory function from 3 to 6 months. This calls for increased remodeling of the alveolar bone compensating for increased attrition of the tooth cusps. In our experiment, BSP was expressed at particularly higher levels in the acellular cementum (Figure 5). BSP has been shown to have a high affinity for collagen and additionally mediates hydroxyapatite formation [45]. BSP in the presence of collagen increases the nucleation potency of hydroxyapatite 10-fold [44,45]. The expression corroborates with the previous studies showing a particular concentration of BSP in the acellular cementum lining the tooth–root surface [32]. In addition, a lack of BSP has been shown to cause periodontal defects in BSP-null mice due to a lack of acellular cementum formation and subsequent attachment [44]. 

OPN antibody has been shown to regulate mineralization in vitro and in vivo and is a constituent of both cementum and alveolar bone [46]. Its role as a potent inhibitor of mineral nucleation, crystal growth, and proliferation has been documented [47]. In our experiment, OPN’s main localization was in the osteoid and in the remodeling region at the calcification front (CZ) border. This expression is consistent with previous studies showing its signal in the mineralization and remodeling regions of the bone [35]. In the metaphysis, the immunostaining was more prominent at the cement lines. These observations are coherent with previous studies with OPN detection in the unmineralized matrix, cellular compartments, and in proteins bound to the mineral phase following immunoblotting of protein extractions [42]. OPN may play a role in regulating the precise rate of crystal nucleation and growth by controlled inhibition. Similarly, the decline in expression at 6 months follows a pattern that is in line with its reduced need with the slowing of the mineralization process corresponding to age and skeletal maturity [46]. 

In the interproximal and furcal bone, the expression pattern showed slowing of the OPN expression from 1 to 6 months. IHC staining by Zhang in 5-week-old mice showed OPN expression was mainly observed in the areas containing newly formed bone in the alveolar process [35]. At the root apex, the positive signals in the cellular cementum remained relatively consistent. Moses et al. obtained similar signal patterns for OPN in bone, dentin, cellular cementum, and periodontal ligament [41]. 

Therefore, all four proteins could be coordinating with each other to allow accurate temporospatial orientation of the mineralized product. The age-dependent changes in the expression of SIBLING proteins may be related to the changes in the need-based functional contribution of progenitor cells. Moreover, the shift in the expression pattern also indicates their possible role in the development and function of these mineralized tissues.

## 5. Conclusions

The expression profile of four SIBLING proteins at three different ages of skeletal development in mice showed variations in staining intensity. The most noticeable IHC staining was found at 3 months of age, which corresponds to the time of greatest conversion of osteoid into the bone in the tibia. The staining intensity in the teeth corresponded to the relative time of the greatest conversion of predentin into dentin. Taken together, the SIBLING proteins were expressed in all age groups albeit at varying levels in different tissues.

## Data Availability

Not applicable.
